# A new chapter for a better *Bioscience Reports*

**DOI:** 10.1042/BSR20211016

**Published:** 2021-05-17

**Authors:** Christopher D.O. Cooper, Weiping Han

**Affiliations:** 1 Deputy Editor-in-Chief (*Bioscience Reports*) and Department of Biological and Geographical Sciences, School of Applied Sciences, University of Huddersfield, Queensgate, Huddersfield, West Yorkshire HD1 3DH, U.K.; 2 Editor-in-Chief (*Bioscience Reports*) and Institute of Molecular and Cell Biology, Agency for Science, Technology and Research (A*STAR), Singapore 138667

**Keywords:** biochemical techniques and resources, biomarkers, cell cycle, genomics, molecular basis of health and disease

## Abstract

As *Bioscience Reports* enters its fifth decade of continuous multidisciplinary life science publishing, here we present a timely overview of the journal. In addition to introducing ourselves and new Associate Editors for 2021, we reflect on the challenges the new Editorial Board has faced and overcome since we took over the editorial leadership in June of 2020, and detail some key strategies on how we plan to encourage more submissions and broader readership for a better and stronger journal in the coming years.

The Biochemical Society is one of the U.K.’s largest single-discipline learned societies, promoting the advancement of the molecular biosciences since 1911. *Bioscience Reports* is published on behalf of the Biochemical Society by Portland Press and is committed to the Society ideals by publishing sound science, providing a suitable home for valid data and findings in the life sciences. We welcome reproducible, appropriately replicated and controlled experiments, with conclusions adequately supported by the presented results. We encourage submissions in all areas of the molecular life sciences, both basic and applied.

*Bioscience Reports* is committed to the Biochemical Society aims of disseminating and sharing scientific knowledge, encouraging discourse and debate amongst scientists. To fully democratise our published research, *Bioscience Reports* has also been committed to full open access since 2012, with all papers published under the most permissive CC BY licence. All journal profits are returned to the Biochemical Society, supporting Society grant-funding and educational charitable endeavours. Since 2020, Portland Press has offered subscribing institutions a combined transformative ‘Read & Publish’ option, facilitating institutions towards full open access publishing [1].

Although *Bioscience Reports* covers a broad range of fields, the journal has maintained an impact factor of 2.942 (issued in 2020), with a 5-year impact factor of 3.112. We feel this puts the journal in an excellent position moving forwards, hopefully allowing us to continue to encourage quality submissions, but to also grow and expand into areas that are currently under-represented, or are becoming of increased topicality.

## Changes

The start of this decade has seen many changes in the scientific community, not least following the global COVID-19 pandemic impacting on the ability of laboratories to conduct research, travel internationally to disseminate findings, and the direct effects of SARS-CoV-2 on scientists themselves, their friends and families. This has also been reflected in research featured in the journal, with a number of studies on SARS-CoV-2 recently published [2–4], including one of our most accessed review articles [5].

**Figure 1:**
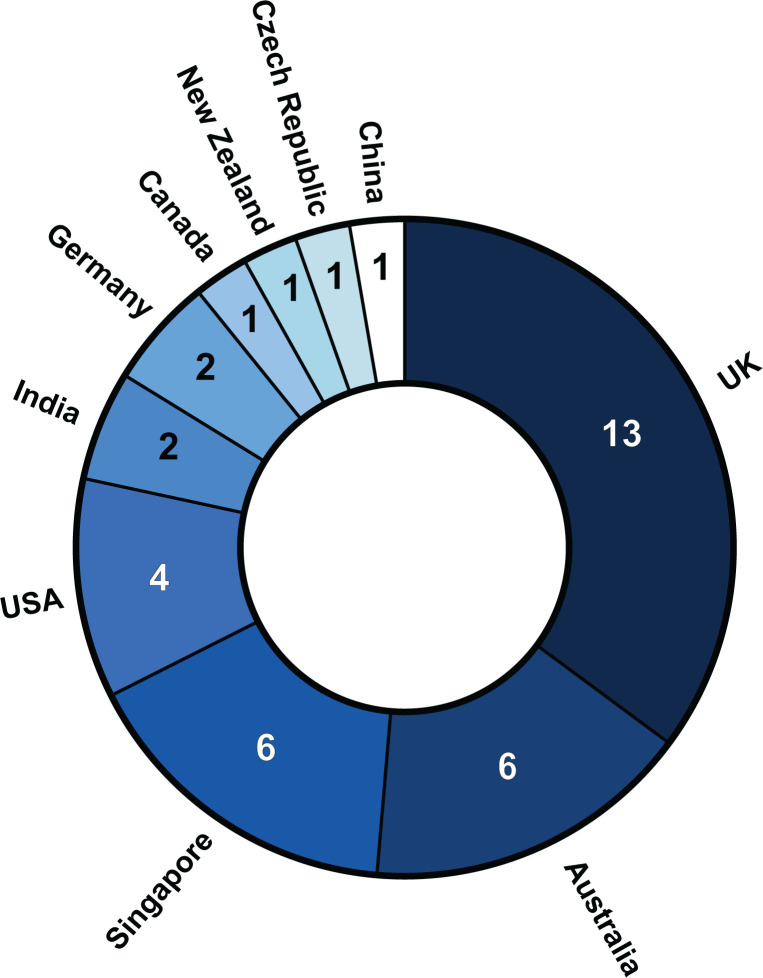
Geographical distribution of the Editorial Board of *Bioscience Reports*

*Bioscience Reports* itself has undergone significant changes in 2020, not least the retirement of our previous Editor-in-Chief Wanjin Hong. We thank Wanjin for his excellent service to the journal for over 10 years, but we take this opportunity to introduce ourselves as the incoming Editor-in-Chief (Weiping Han) and Deputy Editor-in-Chief (Christopher Cooper), with our expertise being in the molecular basis of metabolic disorders and associated complications, and biochemistry and structural molecular biology of genome stability, respectively. To assist in handling increasing numbers of submissions, 2020 also saw a significant expansion of the number of Associate Editors, with 11 recruited to the *Bioscience Reports* Editorial Board (Valerie Chew, Ricardo Correa, Karla Feijs, Lorna Fiedler, Subash Gupta, Michael Huang, Sumit Sahni, Gautam Sethi, Fraser Scott, Jan Skoda and Krishna Rajalingam). This has expanded the subject areas covered by the Editorial Board, including diverse topics such as stem cells, tumour microevironment, cell signalling, ADP-ribosylation, through to chemical biology and drug development. These appointments also diversify the Editorial Board’s geographical distribution (Figure 1), reflecting the international readership of the journal. As the journal continues to grow, we hope to ultimately expand the Editorial Board to approximately 50 Associate Editors, increasing those from under-represented regions (particularly the Americas, Asia and Africa), alongside bringing the journal closer towards gender parity (currently male 65%, female 35%).

We are also broadening our journal scope by encouraging submissions in areas not frequently reported in *Bioscience Reports*, as we outline later. Whilst we encourage submissions reporting new data, we also welcome *in silico* studies. However, here we have recently refined our scope; to strengthen the soundness of ‘omics’ studies analysing existing publicly available datasets, experimental validation prior to peer review may be requested by the Editorial Board, to further increase the soundness of published material.

## Challenges

A recent significant challenge faced by the journal has been the increased prevalence of falsified or fraudulent paper submissions, particularly involving manipulated images such as stock or invented images [6], or duplicated images found both within and between different papers, particularly those pertaining to Western blot, microscopy and flow cytometry data. A particularly worrying trend is the rising frequency of paper mill submissions [7]; the wholesale contract industrialisation or ghostwriting of fabricated papers, or the systematic falsification of research data, or the sharing of once potentially valid (or falsified) data across multiple unrelated publications (exemplified by the so-called ‘tadpole paper mill’, named for the Western blot bands resembling the eponymous under-developed amphibians). Whilst paper mills and methods to detect them have been excellently reviewed by colleagues elsewhere [6,8,9], they remain a clear and present threat to journals in all fields [7]. We especially note trends of paper mill submissions in the field of cancer biomarkers, including studies on small RNAs, bioinformatics analyses of public datasets and single gene knockout studies, as observed elsewhere [10].

We (as Editor-in-Chief and Deputy Editor-in-Chief), the Editorial Board, Portland Press and the Biochemical Society firmly believe in an accurate scientific record, and ensuring the integrity of the material in the journal is of the highest priority. Hence, following a sudden increase in community-driven notifications from PubPeer (and other sources) of published potentially fraudulent and paper mill articles in 2020, we acted rapidly to assess the severity of cases and issued Expressions of Concern to papers where it was felt the issues were both significant and unlikely to have an immediate resolution. This further alerted readers to these papers under investigation, and accordingly published corrections or retracted articles at the earliest timepoint, in accordance with our publishing policies and COPE guidelines. We believe *Bioscience Reports* has taken great steps towards combating such fraudulent submissions, with our other immediate responses including instigating more stringent submission requirements, including the insistence of raw Western blot data and inclusion of institutional email accounts. We continue to mandate ORCID identifiers for corresponding authors, and encourage all authors to include their ORCID ID.

**Figure 2:**
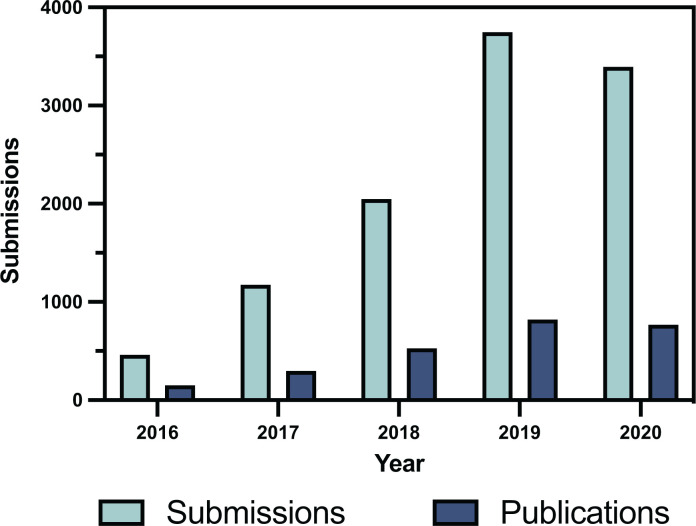
Paper acceptence rate versus submissions in *Bioscience Reports* Data from 2020 includes approximately 300 papers that were rejected before assignment to an Associate Editor, received by *Bioscience Reports* between June and December 2020. Prior to June 2020, papers rejected before assignment to an Associate Editor were not counted in the reported statistics.

In order to better identify issues during peer review, the Editorial Board has also received guidance from experts in the field of image manipulation such as Elisabeth Bik, and we are continually reviewing and updating our editorial processes and policies, with additional checks performed by the Editorial Office upon submission of all articles and again prior to acceptance. Portland Press’ new Data Policy also guides authors in transparent research and data presentation procedures, alongside strengthening our editorial and peer review processes. Whilst *Bioscience Reports* has seen significant growth in the last 5 years, with an over seven-fold increase in submissions compared with 2016 (Figure 2), we expect some of this growth reflects an increase in paper mill submissions. However, we feel the changes outlined here will help to maintain quality, with a current acceptance rate of <25%. Such combined efforts will help to prevent future publication of unsound research, which contributed to the rejection of 385 submitted papers with paper mill, image or authorship concerns in the second half of 2020 alone, illustrating the scale of this issue.

## Looking to the future, and why should authors choose *Bioscience Reports*?

Apart from continuing to ensure the validity and soundness of published articles and rising to counter increases in fraudulent submissions, the Editorial Board hopes to see a continued growth of submitted papers and increase the geographic spread of submissions, yet maintain a suitably high quality. In order to achieve this, we hope to grow the Editorial Board and increase representation and contacts in strategic locations, such as China and North America to handle increased submissions from those regions. Moreover, we hope to especially solicit submissions on a number of topic areas that have traditionally seen fewer submissions to date, to expand the subject base and further reflect the interests of our broad readership. Particular topic areas (with examples of recent published papers) are: protein biochemistry [11], basic molecular biology [12], plant biology [13], microbiology [14], neuroscience [15] and structural biology [16]. By way of approaching this aim we hope to propose a new short protein structural biology report format and we have initiated our first Collection in several years. This Collection will be guest edited by Sven Petersen (Karolinska Institutet, Sweden; Nanyang Technological University, Singapore) and Naama Geva-Zatorsky (Technion, Israel) focusing on the microbiome, with a number of review articles already commissioned.

We feel such broad research topics potentially featuring interdisciplinary studies are of interest to our wider readership, facilitating the exposure of authors’ findings outside of their immediate research field, compared with publishing in specialist journals. This combined with our fully open access publishing model and the values of the journal outlined here, we hope will encourage authors to submit their articles to *Bioscience Reports*.

The journal would be nothing without the publishing team, and we thank all the staff at the Portland Press Editorial Office, particularly Niamh Lynch and Zara Manwaring, the outgoing and incoming Managing Editors, respectively. We also thank the reviewers and readers too for their support in helping to build *Bioscience Reports*, and we look forward to a successful (and post-COVID-19) future.

## References

[1] Legge M. (2019) A refreshed agenda and the ‘open’ road. Biochemist 41, 54–57 10.1042/BIO04104054

[2] Saha P., Banerjee A.K., Tripathi P.P., Srivastava A.K. and Ray U. (2020) A virus that has gone viral: amino acid mutation in S protein of Indian isolate of Coronavirus COVID-19 might impact receptor binding, and thus, infectivity. Biosci. Rep. 40, BSR20201312 10.1042/BSR20201312 32378705 PMC7225408

[3] Bank S., De S.K., Bankura B., Maiti S., Das M. and Khan G.A. (2021) ACE/ACE2 balance might be instrumental to explain the certain comorbidities leading to severe COVID-19 cases. Biosci. Rep. 41, 10.1042/BSR20202014 33442728 PMC7856554

[4] Nizamudeen Z.A., Xu E.R., Karthik V., Halawa M., Arkill K.P., Jackson A.M. et al. (2021) Structural assessment of SARS-CoV2 accessory protein ORF7a predicts LFA-1 and Mac-1 binding potential. Biosci. Rep. 41, 10.1042/BSR20203837 33305306 PMC7796194

[5] Serafim M.S.M., Gertrudes J.C., Costa D.M.A., Oliveira P.R., Maltarollo V.G. and Honorio K.M. (2021) Knowing and combating the enemy: a brief review on SARS-CoV-2 and computational approaches applied to the discovery of drug candidates. Biosci. Rep. 41, 10.1042/BSR20202616 33624754 PMC7982772

[6] Byrne J.A. and Christopher J. (2020) Digital magic, or the dark arts of the 21(st) century-how can journals and peer reviewers detect manuscripts and publications from paper mills? FEBS Lett. 594, 583–589 10.1002/1873-3468.13747 32067229

[7] Else H. and Van Noorden R. (2021) The fight against fake-paper factories that churn out sham science. Nature 591, 516–519 10.1038/d41586-021-00733-5 33758408

[8] Christopher J. (2018) Systematic fabrication of scientific images revealed. FEBS Lett. 592, 3027–3029 10.1002/1873-3468.13201 30047985

[9] Seifert R. (2021) How Naunyn-Schmiedeberg’s Archives of Pharmacology deals with fraudulent papers from paper mills. Naunyn Schmiedebergs Arch. Pharmacol. 394, 431–436 10.1007/s00210-021-02056-8 33547901 PMC7865115

[10] Byrne J.A., Grima N., Capes-Davis A. and Labbe C. (2019) The possibility of systematic research fraud targeting under-studied human genes: causes, consequences, and potential solutions. Biomark. Insights 14, 1177271919829162 10.1177/1177271919829162 30783377 PMC6366001

[11] Lau C.H. and Bolt E.L. (2021) Integration of diverse DNA substrates by a casposase can be targeted to R-loops in vitro by its fusion to Cas9. Biosci. Rep. 41, BSR20203595 10.1042/BSR20203595 33289517 PMC7786333

[12] Idigo N.J., Soares D.C. and Abbott C.M. (2020) Translation elongation factor 1A2 is encoded by one of four closely related eef1a genes and is dispensable for survival in zebrafish. Biosci. Rep. 40, 10.1042/BSR20194191 31950975 PMC6997148

[13] Koenig A.M. and Hoffmann-Benning S. (2020) The interplay of phloem-mobile signals in plant development and stress response. Biosci. Rep. 40, 10.1042/BSR20193329 32955092 PMC7538631

[14] Altamirano S., Jackson K.M. and Nielsen K. (2020) The interplay of phenotype and genotype in Cryptococcus neoformans disease. Biosci. Rep. 40, 10.1042/BSR20190337 33021310 PMC7569153

[15] Maeda T., Inagaki M., Fujita Y., Kimoto T., Tanabe-Fujimura C., Zou K. et al. (2016) ATP increases the migration of microglia across the brain endothelial cell monolayer. Biosci. Rep. 36, 10.1042/BSR20160054 26934979 PMC5293564

[16] Yao L., Swartz P., Hamilton P.T. and Clark A.C. (2021) Remodeling hydrogen bond interactions results in relaxed specificity of Caspase-3. Biosci. Rep. 41, 10.1042/BSR20203495 PMC7846959 33448281

